# Polycythemia and Anemia in Hereditary Hemochromatosis

**DOI:** 10.7759/cureus.7607

**Published:** 2020-04-09

**Authors:** Adnan Aman Khan, Yousaf Hadi, Ayesha Hassan, Justin Kupec

**Affiliations:** 1 Medicine, West Virginia University School of Medicine, Morgantown, USA; 2 Gastroenterology and Hepatology, West Virginia University School of Medicine, Morgantown, USA

**Keywords:** anemia, polycythemia, hereditary hemochromatosis, ferritin, hemoglobin

## Abstract

Introduction

Hereditary hemochromatosis is a syndrome of dysregulated iron homeostasis resulting in the excessive deposition of iron. Hemochromatosis causes pulmonary, pancreatic, and hepatic dysfunction, all of which are risk factors for anemia in the general population. Conversely, iron overload states are thought to predispose to polycythemia. The effect of the homozygosity and heterozygosity of hereditary hemochromatosis-associated genes on hemoglobin levels has not been sufficiently studied.

Materials and methods

We conducted a retrospective cohort study at West Virginia University of all patients who underwent HFE gene analysis and carried the diagnosis of hemochromatosis. Charts were reviewed to identify relevant variables and the patients’ clinical course.

Results

A total of 213 patients were included with 143 male participants (67.13%). The mean age was 53.6 years (SD: 15.2). A total of 108 patients were homozygous for the C282Y mutation. The prevalence of baseline characteristics are as follows: tobacco use 46.3%, chronic obstructive pulmonary disease 16.4%, malignancy 20.1%, cirrhosis 16.8%, anticoagulant use 6.5%, and chronic renal insufficiency 13.1%. The mean hemoglobin of the population was 15.0 mg/dL (SD 2.21). Anemia was seen in 23 patients (10.80%) and 59 patients (27.6%) had polycythemia.

Concurrent malignancy and the presence of chronic renal insufficiency were significantly associated with anemia in both the univariate and multivariate analysis (p-values < 0.001). Patients with homozygosity for C282Y were more likely to receive phlebotomy as compared to other patients. Serum ferritin was not associated with anemia or polycythemia on multivariate analyses (p-values 0.197 and 0.105, respectively).

Conclusion

Despite the high prevalence of comorbidities that are known risk factors for anemia in the general population, few patients with hereditary hemochromatosis develop anemia. Female patients with hereditary hemochromatosis are relatively protected against polycythemia, affecting only one-fourth of all patients with hemochromatosis, with most patients’ serum hemoglobin reported within normal limits.

## Introduction

Hereditary hemochromatosis is an autosomal recessive disorder caused by dysregulated iron homeostasis resulting in excessive iron deposition in parenchymal cells. The organ systems primarily involved include the liver, heart, and endocrine glands (i.e., pancreatic islet cells) [1]. Once excessive deposition ensues, affected parenchymal cells undergo toxic changes resulting in cell death. This may manifest in a wide array of diverse pathologies and clinical syndromes, including, but not limited to, diabetes mellitus, chondrocalcinosis, erectile dysfunction, porphyria cutanea tarda, congestive heart failure, cirrhosis, and hepatocellular carcinoma (HCC) [2].

Multiple genes have been linked with hereditary hemochromatosis, with C282Y homozygosity being the most common genotype associated (80%-85%) [3]. Other genes commonly identified with disease activity include H63D and S65C, among others. Although typically less aggressive, these genes have also demonstrated significant disease activity when paired with C282Y. Phenotypic expression has been reported to occur in approximately 70% of C282Y homozygotes, with fewer than 10% developing severe iron overload and the ensuing clinical manifestations [4-5]. Therapeutic phlebotomy remains the mainstay of treatment, with or without the use of chelating agents.

The organ dysfunction caused by the phenotypic expression of hereditary hemochromatosis is a significant risk factor for anemia of chronic disease. Despite this, previous studies have conjectured that hereditary hemochromatosis may be a significant risk factor for polycythemia due to its effect on iron homeostasis [6]. No definitive association, however, has yet been established. The effect of different genotypes and their phenotypic expressions on anemia and polycythemia has not been sufficiently explored. We, therefore, conducted a retrospective cohort study to further elucidate the effect of hereditary hemochromatosis on anemia and polycythemia, and whether this effect was independent of other known risk factors.

## Materials and methods

A retrospective chart review was conducted for all patients who carried the diagnosis of hemochromatosis using relevant diagnosis codes from the electronic patient database at West Virginia University Medicine (WVUM). All patients who had undergone human homeostatic iron regulator (HFE) gene testing between the years 2010 and 2019 were identified, and their medical charts were reviewed. Patients who carried the clinical diagnosis of hemochromatosis, confirmed to be positive on HFE gene testing, were included. Patients were excluded if they were less than 18 years of age or if no clinical follow-up was available. A total of 213 patients were included in the study and final analysis.

HFE genetic testing is performed by an in-house laboratory at our institution. Testing is conducted for C282Y, H63D, and S65C alleles only. Serum hemoglobin, hematocrit, and iron profiles, including transferrin, total iron-binding capacity (TIBC), and ferritin, are also measured in an in-hospital laboratory 'University Medial Laboratories' that is fully accredited by the College of American Pathologists, The Joint Commission, and the American Association of Blood Banks. The hemoglobin values were reported in gram per deciliter, TIBC in micrograms per deciliter while the serum ferritin levels were reported in nanograms per milliliter.

Our study was reviewed and approved by the Institutional Review Board (IRB) at West Virginia University prior to initiation. A waiver of informed consent was granted for review of medical records.

Data, including demographic characteristics and clinical variables of interest, were extracted by two study personnel. HFE gene test reports were reviewed, and the different genotypes were noted. Serum laboratory hemoglobin levels, hematocrit, ferritin, TIBC, and transferrin saturation values were also collected from patient charts at WVUM. The presence of any obstructive sleep apnea (OSA), chronic obstructive pulmonary disease (COPD), renal insufficiency, concurrent malignancy, along with anticoagulant use, and history of therapeutic phlebotomy was noted.

The World Health Organization (WHO) classification was used to define anemia and polycythemia as follows. Males with hemoglobin values less than 13 g/dL were classified as anemic, and those with values greater than 16.5 g/dL or hematocrit greater than 49 percent were categorized as having polycythemia [7-8]. Similarly, females with hemoglobin less than 12 g/dL were classified as being anemic and those with values greater than 16.0 g/dL or hematocrit greater than 48 percent were considered to have polycythemia.

Statistical analysis was performed using statistical software R (2019, version 3.6.2) [9]. Rates of anemia and polycythemia were compared between different genotypic variants. Univariate analysis was conducted using t-tests and chi-square tests for continuous and categorical variables, respectively. Variables were then incorporated into a multivariate logistic regression model to assess for independent associations and control for confounders. P-values less than 0.05 were considered significant for the purposes of this study.

## Results

A total of 213 participants fulfilled the criteria for the study and were included in the final analysis. The mean age of the study participants was 53.6 years (±15.2); 143 (67.13%) participants were male. The mean hemoglobin value of the study population was 15.0 mg/dL (±2.21). The prevalence of tobacco use was 46.3%, and 16.4% of participants were diagnosed with chronic obstructive pulmonary disease (COPD). The baseline characteristics of our study population are described in Table 1.

**Table 1:** Characteristics of the study population

Variable	Total Population	Other Genotypes	Homozygous C282Y
Mean Age (in years)	53.61 (±16.20)	54.50 (±14.52)	52.73 (±15.78)
Male Gender	143 (67.3%)	68 (64.76%)	65 (60.19%)
C282Y Homozygosity (n)	108		
C282Y Heterozygosity (n)	1		
H63D Homozygosity (n)	18		
H63D/C282Y (n)	39		
S65C/C282Y (n)	2		
H63D Heterozygosity (n)	46		
Smoking History	99 (46.48%)	52 (49.52%)	47 (43.52%)
Obstructive Sleep Apnea	32 (15.02%)	16 (15.24%)	16 (14.81%)
Anticoagulant Use	14 (6.57%)	7 (6.67%)	7 (6.48%)
Chronic Renal Insufficiency	28 (13.15%)	14 (13.33%)	14 (12.96%)
Chronic Obstructive Pulmonary Disease	32 (15.02%)	16 (15.24%)	16 (14.81%)

The prevalence of cirrhosis in our study population was 16.8% and 20.1% of patients had a concurrent diagnosis of malignancy. Anticoagulant use was present in 6.5% of patients and 13.1% had been diagnosed with chronic renal insufficiency. One hundred and sixteen patients (54.46% of the patient population) were receiving therapeutic phlebotomies.

Of the total study population, 23 patients had hemoglobin levels consistent with anemia as defined above (10.80%) while 59 patients had polycythemia (27.70%). Mean hemoglobin was higher in males as compared to females (15.56% vs 14.09%, p-value < 0.001). Univariate tests revealed no difference between the mean hemoglobin levels of patients who were homozygous for C282Y, patients who were homozygous for H63D, or patients who were heterozygous for the mutations (p-values > 0.05).

Univariate analyses revealed that the presence of concurrent malignancy (p-value 0.0001) and chronic renal insufficiency (p-value 0.0001) were significantly associated with the presence of anemia. Male gender was significantly associated with the presence of polycythemia (p-value 0.0002). Patients who were homozygous for C282Y were more likely to receive phlebotomy as compared to other genotypes with a p-value of less than 0.001 (Table 2).

**Table 2:** Univariate analysis comparing homozygous C282Y with other genotypes

Variable	Homozygous C282Y	Other Genotypes	P-Values
Ferritin (ng/mL)	834.40 (±79.13)	548.98 (±757.39)	0.083
Transferrin saturation	55.68 (30.64%)	50.60 (37.54%)	0.31
Anemia	7 (6.48%)	16 (15.23%)	0.07
Polycythemia	26 (24.07%)	33 (31.42%)	0.29
Phlebotomy	74 (68.52%)	42 (40%)	< 0.0001

**Figure 1:**
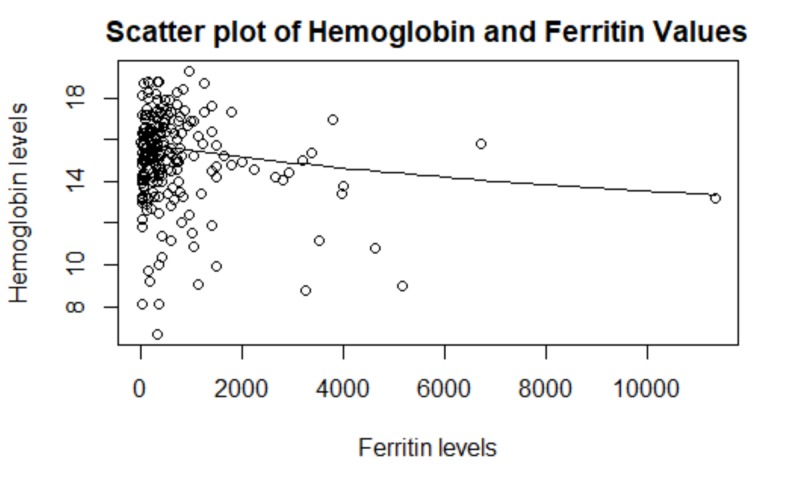
Scatter plot of hemoglobin and ferritin values

Multivariable logistic regression (incorporating concurrent malignancy, genotype status, chronic kidney disease, age, gender, smoking status, and COPD) revealed that chronic renal insufficiency (odds ratio (OR) 14.19, 95% confidence interval (CI) 4.17-55.90; p-value 0.0005) and presence of concurrent malignancy (OR 10.34, 95% CI 3.15-39.29; p-value 0.0002) were significantly associated with anemia. Female gender was protective against polycythemia (OR: 0.289 95% CI: 0.13-0.61, p-value 0.001). Serum ferritin was not associated with anemia or polycythemia on multivariate analyses (p-values of 0.197 and 0.105, respectively). A scatterplot of hemoglobin values plotted against ferritin is depicted in Figure 1. 

## Discussion

Iron homeostasis involves a complex interplay of multiple cellular pathways mediated by hepcidin, a peptide synthesized by the liver [10]. When bound to ferroportin, the transmembrane iron-transport receptor, hepcidin activates signaling pathways that result in endocytosis and degradation of the iron-transport receptor. As a result, the intracellular uptake of iron is inhibited. Hepcidin synthesis is regulated by levels of serum and stored iron through sophisticated feedback mechanisms. In hereditary hemochromatosis, genetic mutations involving the hepcidin gene results in elevated serum iron and ferritin levels as a consequence of dysregulated iron homeostasis.

Polycythemia has been previously explored in various studies in the context of hereditary hemochromatosis [6,11]. Polycythemia in this setting is thought to be a consequence of elevated transferrin saturation levels, resulting in increased iron uptake by erythroid precursor cells. Intracellular iron is then utilized by these precursor cells to increase red blood cell synthesis. Previous studies have also suggested that utilization of non-transferrin bound iron may occur by erythroid precursor cells through transferrin-independent pathways [12]. Heme synthesis, in normal erythroid cells, is regulated through the feedback inhibition of aminolevulinic acid (ALA) synthase and ALA dehydrase, among other proposed mechanisms [13]. There is no current literature describing the utilization of and the mechanisms by which iron metabolism differs in hereditary hemochromatosis, therefore, it is certainly possible that significant differences may be present from normal erythroid cells.

Although an elevated hemoglobin value is, at times, considered to be a feature of hereditary hemochromatosis, we found a mean hemoglobin value of 15.0 mg/dL in our patient population. Only one-fourth of study participants met the WHO cutoff for polycythemia, even in the setting of exceedingly elevated serum ferritin levels. The common misassociation of hemochromatosis with polycythemia may lead to lower suspicion for hemochromatosis by providers in patients with anemia or normal hemoglobin, thus leading to missed diagnoses [14]. Our data, as well as the limited current available data on hemoglobin parameters, as discussed below, does not support a high prevalence of polycythemia in these individuals.

Limited published data on blood erythrocyte parameters in patients with hereditary hemochromatosis is available in the current literature. Beutler et al. report only a slightly increased mean hemoglobin level in patients with hereditary hemochromatosis screened in the ambulatory setting [10]. Asif et al. observed a median hemoglobin level of 15.5 mg/dL in their homozygous C282Y population, which is closely reflective of the hemoglobin values reported by our study [11]. Another study by Barton et al. reports that only patients with C282Y homozygosity (and not other genotypic variants) have higher mean hemoglobin values when compared to healthy matched controls; the mean hemoglobin levels of their untreated homozygous C282Y probands was reported to be 15.0 mg/dL (±1.3), which also closely matches that reported in our study [6]. The percentage of participants meeting the criteria for polycythemia was not discussed.

It can be deduced that although it may offer a degree of relative protection against anemia, hereditary hemochromatosis does not present with polycythemia in a majority of patients. We did not find any association between ferritin levels and polycythemia or anemia in our cohorts, which may suggest that the iron overload state in these patients may not be reflected by changes in the hemoglobin profile.

Our study is limited by its retrospective nature. Only the C282Y, H63D, and S65C genes were reported at our facility. However, with a larger sample size than the previous limited data on the subject, and after controlling for all possible confounders, we have found that patients with hereditary hemochromatosis mostly present with normal hemoglobin values, with a low prevalence of anemia or polycythemia.

## Conclusions

Despite the high prevalence of medical comorbidities that are known risk factors for the development of anemia in the general population, very few study participants with hereditary hemochromatosis developed anemia. Female patients were relatively protected against the development of polycythemia, affecting only one-fourth of all patients. Serum ferritin levels were not associated with anemia or polycythemia, with most patients’ serum hemoglobin falling within normal limits.
